# Aristolochic Acid-Induced Autophagy Promotes Epithelial-to-Myofibroblast Transition in Human Renal Proximal Tubule Epithelial Cells

**DOI:** 10.1155/2017/9596256

**Published:** 2017-10-18

**Authors:** Yu-Lin Man, Hong-Liang Rui, Yi-Pu Chen, Guo-qin Wang, Li-Jun Sun, Hong Cheng

**Affiliations:** Division of Nephrology, Beijing Anzhen Hospital, Capital Medical University, Beijing 100029, China

## Abstract

Autophagy plays an essential role in cellular homeostasis in kidney. Previous studies have found that aristolochic acid (AA) can induce autophagy of renal tubular epithelial cells and epithelial-to-myofibroblast transition (EMT). However, the relationship between AA-induced autophagy and EMT is unclear. Our results showed that, after AA stimulation, the appearance of autophagy preceded EMT. Autophagy of HKC cells began to increase gradually from the 3rd hour, reached the peak at 12th hour, and then weakened gradually until 36th hour; the EMT process of HKC continued to increase from 6th hour to 36th hour after AA stimulation. The enhancement of autophagy using autophagy inducers, rapamycin or serum-free medium, led to an aggravation of EMT and upregulated expression of fibronectin, a component of extracellular matrix, in AA-treated HKC cells. In contrast, the inhibition of autophagy by autophagy inhibitor, 3-methyladenine, or by knockdown of* Beclin 1* led to an attenuation of EMT and downregulated expression of fibronectin in AA-treated HKC cells. Taken together, our study suggests that, after AA stimulation, two types of cell responses of HKC cells, autophagy and EMT, will successively appear, and autophagy can promote EMT of HKC.

## 1. Introduction

Aristolochic acid (AA) is a component of* Aristolochia* genus of plants [1, 2]. AA is capable of inducing aristolochic acid nephropathy (AAN) including so-called Balkan endemic nephropathy, which is often associated with urothelial malignancies [1–3]. Chronic aristolochic acid nephropathy (CAAN) is pathologically characterized by an extensive interstitial fibrosis associated with ischemic glomeruli and tubular atrophy [4]. Both* in vitro* and* in vivo* studies have shown that renal tubular epithelial cells are one of the direct targets of AA action [5, 6]. The toxic effects of AA on proximal tubular epithelial cells can induce the cytokine secretion promoting extracellular matrix (ECM) synthesis and epithelial-to-myofibroblast transition (EMT) [4, 5].

Epithelial-to-myofibroblast transition is characterized by that tubular epithelial cells lose their epithelial cell properties, acquire mesenchymal properties, and eventually convert into the phenotype of myofibroblasts [7, 8]. Myofibroblasts will secrete ECM components including collagen type I and type III and fibronectin, which accumulation will lead to renal interstitial fibrosis [9, 10]. In addition, recent studies have also reported the crucial role of partial EMT in the renal interstitial fibrosis [8, 11]. Partial EMT is an intermediate state characterized by expressing both biomarkers of epithelial and mesenchymal cells, which relays signals to the interstitium to promote myofibroblast activation and fibrogenesis without directly generating interstitial myofibroblasts [8, 11]. We and others researchers have reported that EMT can be observed in the rodent models of CAAN and cultured renal tubular epithelial cells stimulated by AA [5, 12].

Autophagy is traditionally known as a cellular process of “self-digestion,” which is an adaptive responder of cells to nutrient deprivation to maintain cellular homeostasis [13]. It is also a conserved cellular degradative process important for cellular survival [14]. The overall procedure of autophagy can be divided into three phases: firstly, sequestration, in which cellular components are captured by double-bilayer membranes to form the autophagosome; secondly, transport of the autophagosome to the lysosome; and, thirdly, degradation or maturation, which involves vesicle fusion and mixing of autophagosomal contents with lysosomal hydrolases to eventually release degraded byproducts back into the cytosol through membrane permeases [14, 15]. Now numerous evidences have indicated that autophagy plays an essential role in kidney health, aging, and disease [13, 15]. Autophagy is regulated by a core group of conserved autophagy-related (Atg) genes that were originally identified in yeast [16]. Among Atg proteins, microtubule-associated protein light chain 3 (LC3), the mammalian ortholog of Atg8, is a crucial component of the autophagosomal membrane and plays an important role in elongation and closure of cargo-containing autophagosomes [17]. Beclin1, a mammalian ortholog of the Atg 6, plays a pivotal role in the autophagic initiation process that involves nucleation of the autophagic vesicle [18]. Thus LC3-II and Beclin 1 are often used as biomarkers of autophagy [19, 20].

Recent studies have shown that AA-induced autophagy is closely related to cell apoptosis in the renal tubular epithelial cells [19, 20]. However, the relationship between AA-induced autophagy and EMT in renal tubular epithelial cells has not been reported. In this study, we researched the relationship between these two types of cell responses. Our results showed that autophagy took place earlier than EMT occurrence in human renal proximal tubular cell line (HKC) during AA stimulation. In addition, inhibition of autophagy could alleviate EMT, and, on the contrary, enhancement of autophagy could aggravate EMT in the AA-treated HKC. Our results suggest that the AA-induced autophagy is able to promote EMT in renal tubular epithelial cells, and there is a close relationship between AA-induced autophagy and EMT.

## 2. Materials and Methods

### 2.1. Cell Culture

The human proximal tubular epithelial cell line (HKC), which was originally developed by Professor L. C. Racusen, was kindly provided by Professor F. L. Zheng [4]. HKC cells were incubated in DMEM/F12 medium (Life Technologies, USA) containing 10% inactivated fetal bovine serum (Life Technologies, USA), 100 U/ml penicillin, and 100 mg/ml streptomycin at 37°C, 5% CO_2_. In the experiments with 3-methyladenine (3-MA, Sigma-Aldrich, USA) or rapamycin (Sigma-Aldrich, USA), cells were pretreated for 60 min with 3-MA or rapamycin and then coincubated with AA (Sigma, USA). In the experiments of serum-free medium, HKC cells were incubated in DMEM/F12 medium without fetal bovine serum. After 12 h or 24 h of incubation, the cells were harvested for real-time quantitative polymerase chain reaction (PCR) analysis or Western blot assay, respectively.

### 2.2. MTT Colorimetric Assay

Cellular viability was determined by MTT colorimetric assay. HKC cells were seeded in 96-well culture plate and stimulated with AA at various concentrations (0 to 30 *μ*mol/L). After 36 h of stimulation, cells were incubated with 0.5 mg/ml MTT (Sangon Biotech, China) for 4 h at 37°C, and then the supernatants were discarded. The MTT-formazan crystals formed in the metabolically viable cells were dissolved in dimethyl sulphoxide (DMSO). The absorbance at 570 nm was measured with a microplate reader (Bio-TEK Elx800, USA).

### 2.3. Lactate Dehydrogenase Release Test

To evaluate the toxicity of AA on HKC cells, the release test of lactate dehydrogenase (LDH), a cytoplasmic enzyme, was performed. The treatment of HKC cells with AA was the same as that in MTT colorimetric assay. The activity of LDH in the cell culture supernatants was measured with a CytoTox 96® Non-Radioactive Cytotoxicity Assay Kit (Promega, USA) and the LDH release rate (%) was calculated according to the instruction manual of the kit.

### 2.4. Cell Transfection

HKC cells were incubated to 70–80% confluence in six-well culture plate and then transiently transfected with 4 *μ*l Beclin 1 siRNA (Santa Cruz, sc-29797) or 4 *μ*l control siRNA-A (Santa Cruz, sc-37007) by using Lipofectamine 2000 (Life Technologies, USA) according to the manufacturer's instruction. After that, the transfected HKC cells were cultured for 24 h and then incubated with or without 10 *μ*mol/L AA. After 12 h and 36 h of incubation, the transfected cells were harvested for real-time quantitative PCR analysis and Western blot assay, respectively.

### 2.5. Reverse Transcription and Real-Time Quantitative PCR Analysis

Total RNA was extracted using Trizol reagent (Life Technologies, USA) according to the manufacturer's instruction. The reverse transcription from 2 *μ*g RNA to cDNA was implemented with Moloney murine leukemia virus reverse transcriptase (Promega, USA). Quantitative real-time PCR was performed using SYBR Green Real-time PCR Master Mix (TOYOBO, Japan). The gene-specific primers are listed in Table 1. The *β*-actin was set as the internal control gene. The gene expression was calculated by the following formula: 2^−(target  gene  Ct-*β*-actin  Ct)^ × 10^3^, where Ct is threshold cycle number. The reverse transcription and real-time quantitative PCR were repeated at least in five times independently.

### 2.6. Western Blot Assay

Total protein lysates were extracted from the HKC cells using RIPA lysis buffer (CW Biotec, China). Protein samples were sonicated five times for 1 sec each, centrifuged at 13000 rpm for 20 min at 4°C, and then boiled for 5 min. Equal amounts of protein samples were separated by 10% sodium dodecyl sulphate-polyacrylamide gel electrophoresis (SDS-PAGE) and transferred to nitrocellulose membranes (General Electric Co, USA). After blocking with 5% skim milk in phosphate-buffered saline with 0.1% Tween 20 for 1 h, the membranes were incubated with primary antibody at 4°C overnight and then with secondary antibody for 1 h at room temperature. The primary and secondary antibodies are listed in Table 2. The bound antibodies were visualized by Odyssey Infrared Imaging System (LI-COR Biosciences, USA). *β*-Actin was set as an internal control. The relative expression level of the target protein was displayed as a ratio of target protein/*β*-actin protein. All the assays were repeated at least in five times independently.

### 2.7. Statistical Analysis

The values are represented as means ± SD. One-way ANOVA was used to test the differences among groups. Statistical significance was defined as *P* < 0.05.

## 3. Results

### 3.1. The Determination of AA Concentration in Cell Experiments

To observe AA-induced autophagy and EMT of HKC cells, the appropriate concentration of AA for stimulating cells should be determined. At such a concentration, AA does not affect cellular viability and has also no cytotoxicity.

**Figure 1:**
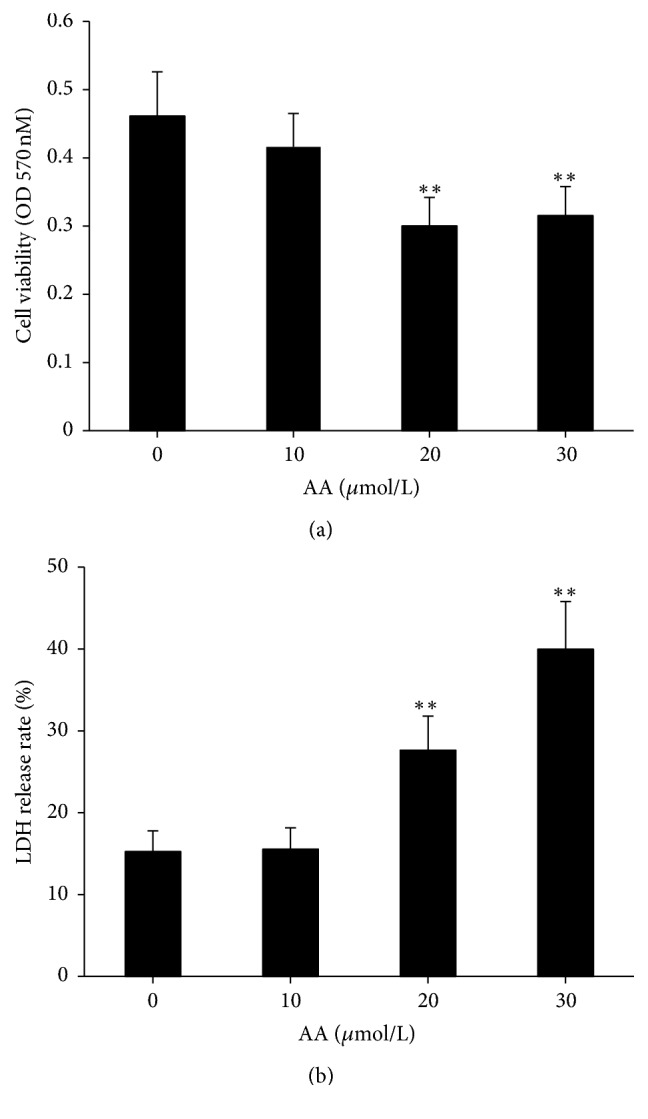
*Effects of AA on cellular viability and cytotoxicity of HKC cells.* (a) Viability of HKC cells was determined with MTT assay after cells were treated with various concentrations of AA. (b) Cytotoxicity of HKC cells was determined with LDH release test after cells were treated with various concentrations of AA. Values are represented as mean ± SD (*n* = 8). ^*∗∗*^*P* < 0.01 compared with control group.

The effects of AA on HKC cellular viability and cytotoxicity were detected with MTT colorimetric assay and LDH release test, respectively. Cells were treated with AA of different concentrations (0, 10, 20, and 30 *μ*mol/L) for 36 h. Results showed that 20 and 30 *μ*mol/L AA significantly decreased cellular viability of HKC compared with 0 *μ*mol/L AA (*P* < 0.01), while 10 *μ*mol/L AA had no effect on cellular viability (Figure 1(a)). Similarly, 10 *μ*mol/L AA had also no cytotoxic effect on HKC cells, but 20 and 30 *μ*mol/L AA significantly increased the LDH release rate compared with 0 *μ*mol/L AA (*P* < 0.01) (Figure 1(b)).

According to the above results of MTT colorimetric assay and LDH release test, 10 *μ*mol/L was chosen as the concentration of AA stimulating cells in all experiments.

### 3.2. Temporal Dynamic Changes of AA-Induced Autophagy and EMT in HKC Cells

To investigate the temporal dynamic changes of AA-induced autophagy and EMT in HKC cells, the expression of biomarkers of autophagy and EMT at different time was detected. The biomarkers of autophagy using in this study were LC3-II and Beclin 1, and the biomarkers of EMT were E-cadherin and *α*-SMA.

**Figure 2:**
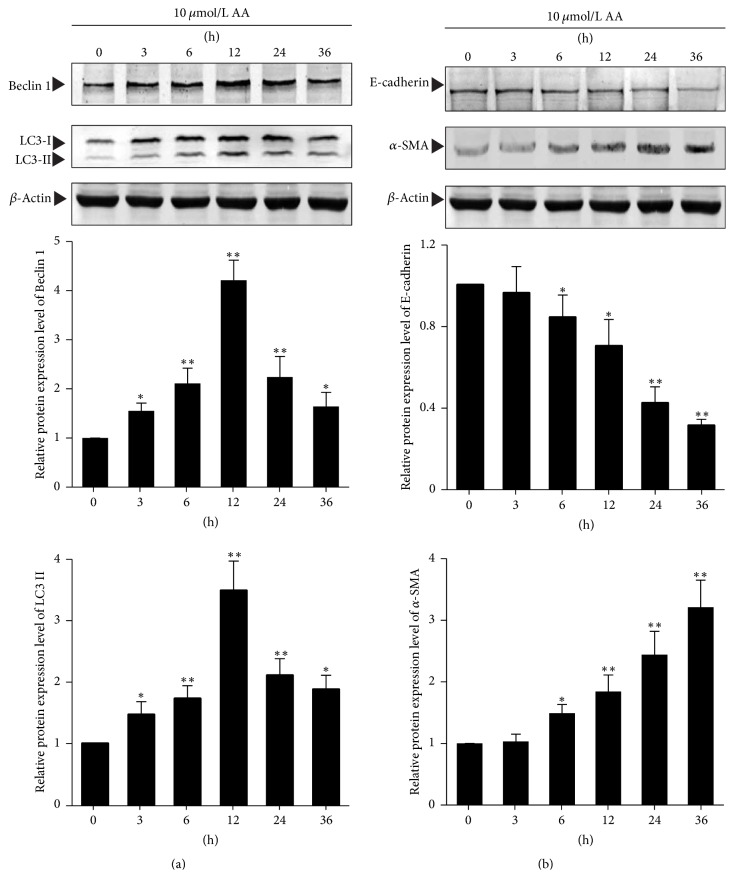
*Temporal dynamic changes of AA-induced autophagy and EMT in HKC cells.* HKC cells were incubated with 10 *μ*mol/L AA for different durations. (a) and (b) After stimulation with AA, HKC cells were harvested to detect the protein expression of LC3-II, Beclin 1, E-cadherin, and *α*-SMA by Western blot assay. The relative protein expression level is expressed as the ratio of target protein/*β*-actin. Experiments were repeated 5 times independently. Values are represented as mean ± SD (*n* = 5). ^*∗*^*P* < 0.05, ^*∗∗*^*P* < 0.01 compared with control group.

As shown in Figure 2, after HKC cells were stimulated with 10 mmol/L AA, the relative protein expression levels of LC3-II and Beclin 1 were significantly increased from 3rd hour to 36th hour compared with that at 0 hour (*P* < 0.05 or *P* < 0.01), and the expression level reached peak at 12th hour and then gradually weakened (Figure 2(a)). In addition, after HKC cells were stimulated with 10 mmol/L AA, the relative protein expression levels of E-cadherin were continuously decreased, while the relative protein expression levels of *α*-SMA were continuously increased from 6th hour to 36th hour compared with those at 0 hour (*P* < 0.01) (Figure 2(b)). These results suggest that autophagy and EMT of HKC cells both occurred after AA stimulation, but the temporal dynamic changes of their expression intensity were different.

### 3.3. Enhancement of Autophagy Promotes EMT in AA-Treated HKC Cells

**Figure 3:**
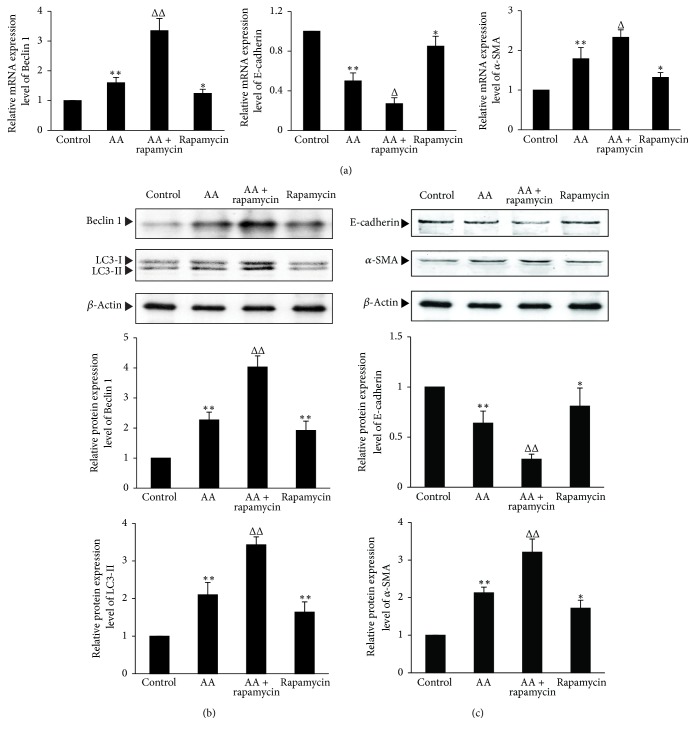
*The effects of enhancing autophagy by rapamycin on EMT in the AA-treated HKC cells.* HKC cells were incubated in medium, medium containing 10 *μ*mol/L AA, medium containing 10 *μ*mol/L rapamycin, and medium containing 10 *μ*mol/L AA with 10 *μ*mol/L rapamycin, respectively. (a) After 12 h incubation, cells were harvested and then mRNA expression levels of Beclin1, E-cadherin, and *α*-SMA were measured by real-time quantitative PCR. (b) and (c) After 24 h incubation, HKC cells were harvested to detect the protein expression levels of LC3-II, Beclin 1, E-cadherin, and *α*-SMA by Western blot assay. The relative protein expression level is expressed as the ratio of target protein/*β*-actin. Experiments were repeated 5 times independently. Values are represented as mean ± SD (*n* = 5). ^*∗∗*^*P* < 0.01 versus control group. ^Δ^*P* < 0.05, ^ΔΔ^*P* < 0.01 versus AA group. ^*∗*^*P* < 0.05 versus control group.

To test the effect of autophagy on EMT in AA-treated HKC cells, an autophagy inducer, rapamycin, was used for the experiment. As shown in Figure 3, compared with AA group, rapamycin further enhanced AA-induced LC3-II protein expression (*P* < 0.01), which was coupled with further enhanced Beclin 1 mRNA and protein expression (*P* < 0.01) (Figures 3(a) and 3(b)). In addition, compared with AA group, rapamycin upregulated AA-induced mRNA and protein expression of *α*-SMA (*P* < 0.05 or *P* < 0.01), which was coupled with further downregulated mRNA and protein expression of E-cadherin (*P* < 0.05 or *P* < 0.01) (Figures 3(a) and 3(c)). These results suggest that induction of autophagy can significantly increase EMT in AA-treated HKC cells.

**Figure 4:**
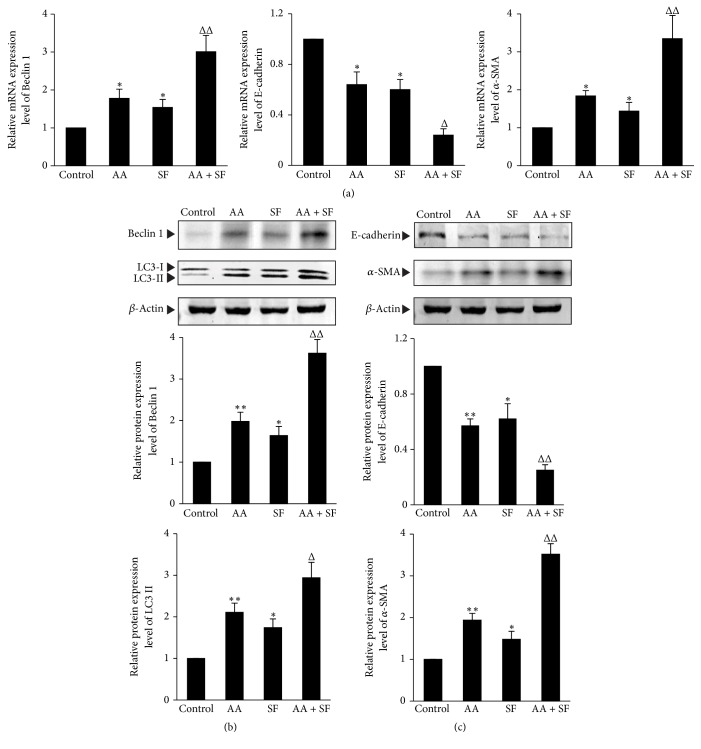
*The effects of enhancing autophagy by serum-free medium on EMT in the AA-treated HKC cells.* HKC cells were incubated in medium, medium containing 10 *μ*mol/L AA, serum-free (SF) medium, and SF medium containing 10 *μ*mol/L AA, respectively. (a) After 12 h incubation, cells were harvested and then mRNA expression levels of Beclin 1, E-cadherin, and *α*-SMA were measured by real-time quantitative PCR. (b) and (c) After 24 h incubation, HKC cells were harvested to detect the protein expression levels of LC3-II, Beclin 1, E-cadherin, and *α*-SMA by Western blot assay. The relative protein expression level is expressed as the ratio of target protein/*β*-actin. Experiments were repeated 5 times independently. Values are represented as mean ± SD (*n* = 5). ^*∗*^*P* < 0.05, ^*∗∗*^*P* < 0.01 versus control group. ^Δ^*P* < 0.05, ^ΔΔ^*P* < 0.01 versus AA group.

To confirm the experimental results of rapamycin, the experiment of serum-free (SF) medium, another biological inducer of autophagy, was also implemented. Similarly, SF medium significantly enhanced the AA-induced autophagy of HKC cells, which was shown as further enhancement of LC3-II protein expression and Beclin 1 mRNA and protein expression (*P* < 0.05 or *P* < 0.01 versus AA group) (Figures 4(a) and 4(b)). In addition, SF medium also significantly enhanced the AA-induced ECM of HKC cells, which was displayed as further upregulation of *α*-SMA mRNA and protein expression (*P* < 0.01 versus AA group) and further downregulation of E-cadherin mRNA and protein expression (*P* < 0.05 or *P* < 0.01 versus AA group) (Figures 4(a) and 4(c)). The above experimental results, taken together, suggest that enhancement of autophagy can significantly promote EMT in AA-treated HKC cells.

### 3.4. Inhibition of Autophagy Attenuates EMT in AA-Treated HKC Cells

**Figure 5:**
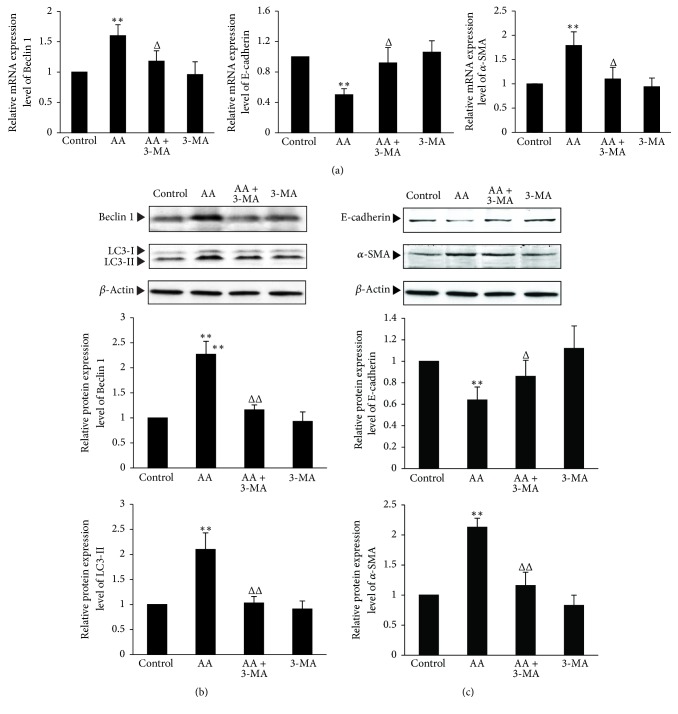
*The effects of inhibiting autophagy by 3-MA on EMT in AA-treated HKC cells.* HKC cells were incubated in medium, medium containing 10 *μ*mol/L AA, medium containing 5 mmol/L 3-MA, and medium containing 10 *μ*mol/L AA with 5 mmol/L 3-MA, respectively. (a) After 12 h incubation, cells were harvested and then mRNA expression levels of Beclin 1, E-cadherin, and *α*-SMA were measured by real-time quantitative PCR. (b) and (c) After 24 h incubation, HKC cells were harvested to detect the protein expression levels of LC3-II, Beclin 1, E-cadherin, and *α*-SMA by Western blot assay. The relative protein expression level is expressed as the ratio of target protein/*β*-actin. Experiments were repeated 5 times independently. Values are represented as mean ± SD (*n* = 5). ^*∗∗*^*P* < 0.01 versus control group. ^Δ^*P* < 0.05, ^ΔΔ^*P* < 0.01 versus AA group.

To test whether the inhibition of autophagy can attenuate EMT in AA-treated HKC cells, 3-MA, an inhibitor of autophagy, was used for the experiment. As shown in Figure 5, compared with AA group, 3-MA significantly decreased AA-induced LC3-II protein expression (*P* < 0.01), which was coupled with the significantly decreased Beclin 1 mRNA and protein expression (*P* < 0.05 or *P* < 0.01) (Figures 5(a) and 5(b)). In addition, compared with AA group, 3-MA significantly downregulated the AA-induced mRNA and protein expression of *α*-SMA (*P* < 0.05 or *P* < 0.01), which was coupled with the significantly upregulated mRNA and protein expression of E-cadherin (*P* < 0.05) (Figures 5(a) and 5(c)). These results suggest that the inhibition of autophagy can significantly weaken EMT in AA-treated HKC cells.

**Figure 6:**
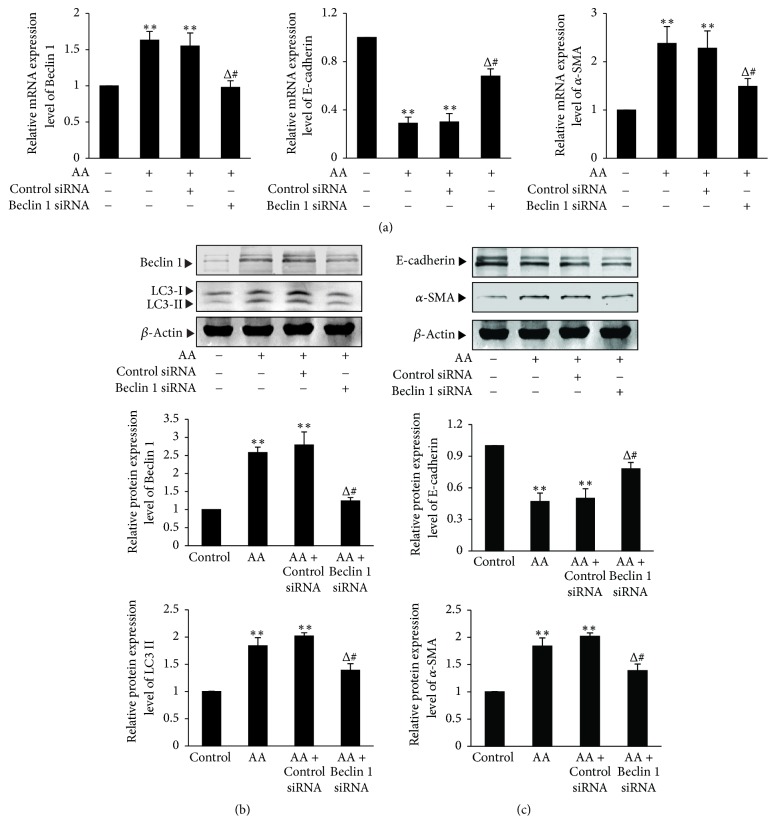
*The effects of inhibiting autophagy by Beclin 1 gene knockdown on EMT in AA-treated HKC cells.* HKC cells were transiently transfected with Beclin 1 siRNA or control siRNA. Afterwards, the experiments were divided into the following 4 groups: HKC cells in medium; HKC cells in medium containing 10 *μ*mol/L AA; HKC cells transfected with control siRNA in medium containing 10 *μ*mol/L AA; HKC cells transfected with Beclin 1 siRNA in medium containing 10 *μ*mol/L AA. (a) After 12 h incubation, cells were harvested and then mRNA expression levels of Beclin 1, E-cadherin, and *α*-SMA were measured by real-time quantitative PCR. (b) and (c) After 24 h incubation, HKC cells were harvested to detect the protein expression levels of LC3-II, Beclin 1, E-cadherin, and *α*-SMA by Western blot assay. The relative protein expression level is expressed as the ratio of target protein/*β*-actin. Experiments were repeated 5 times independently. Values are represented as mean ± SD (*n* = 5). ^*∗∗*^*P* < 0.01 versus control group; ^Δ^*P* < 0.05 versus AA group; ^#^*P* < 0.05 versus AA + control siRNA group.

To confirm the experimental results of 3-MA, the experiment of Beclin 1 gene knockdown was also performed. Beclin 1 siRNA and control siRNA were transfected into HKC cells, respectively. Real-time quantitative PCR analysis and Western blot assay revealed that the Beclin 1 mRNA and protein expression was significantly downregulated in Beclin 1 siRNA group (*P* < 0.05) but not changed in control siRNA-A group (*P* > 0.05), compared with control group (Figures 6(a) and 6(b)). The results suggest the siRNA transfection is successful.

Results showed that Beclin 1 siRNA transfection significantly downregulated the AA-induced protein expression of LC3-II compared with AA group (*P* < 0.05) (Figure 6(b)). In addition, Beclin 1 gene knockdown also significantly downregulated the AA-induced mRNA and protein expression of *α*-SMA (*P* < 0.05) and significantly upregulated the AA-reduced mRNA and protein expression of E-cadherin compared with AA group (*P* < 0.05) (Figures 6(a) and 6(c)). All of the above results suggest that the inhibition of autophagy can significantly attenuate EMT in AA-treated HKC cells.

### 3.5. Effects of Autophagy on Expression of Fibronectin in AA-Treated HKC Cells

**Figure 7:**
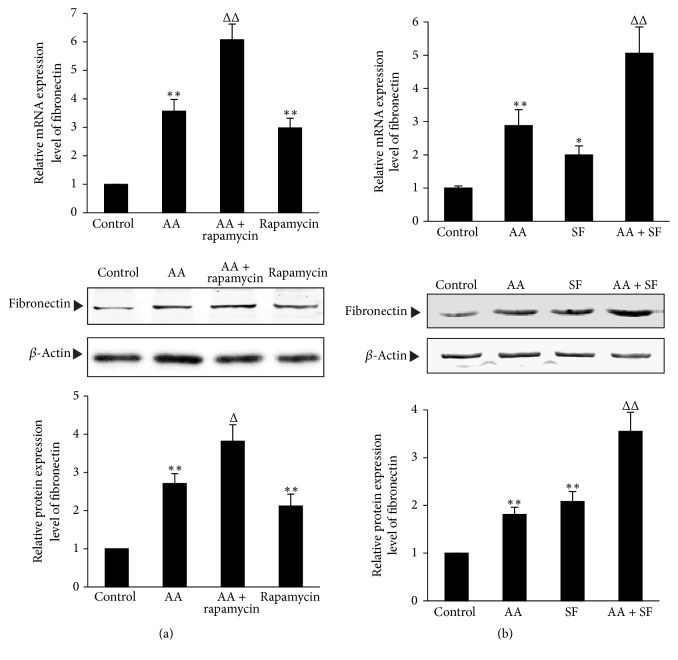
*The effects of enhancing autophagy on fibronectin expression in AA-treated HKC cells.* (a) HKC cells were incubated in medium, medium containing 10 *μ*mol/L AA, medium containing 10 *μ*mol/L rapamycin, and medium containing 10 *μ*mol/L AA with 10 *μ*mol/L rapamycin, respectively. (b) HKC cells were incubated in medium, medium containing 10 *μ*mol/L AA, serum-free (SF) medium, and SF medium containing 10 *μ*mol/L AA, respectively. After 12 h (for mRNA detection) and 36 h (for protein measurement) incubation, the mRNA and protein expression of fibronectin were determined by real-time quantitative PCR and Western blot assay, respectively. The relative protein expression level is expressed as the ratio of target protein/*β*-actin. Experiments were repeated 5 times independently. Values are represented as mean ± SD (*n* = 5). ^*∗*^*P* < 0.05, ^*∗∗*^*P* < 0.01 versus control group. ^Δ^*P* < 0.05, ^ΔΔ^*P* < 0.01 versus AA group.

**Figure 8:**
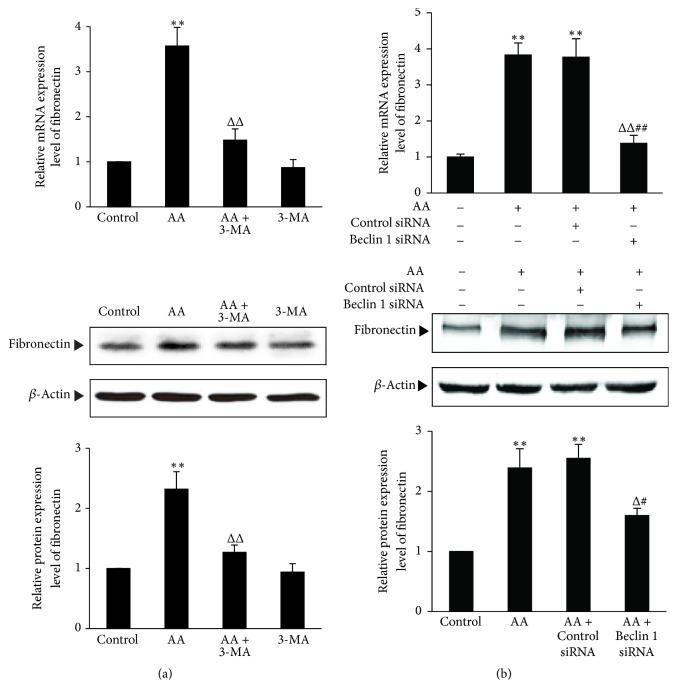
*The effects of inhibiting autophagy on fibronectin expression in AA-treated HKC cells.* (a) HKC cells were incubated in medium, medium containing 10 *μ*mol/L AA, medium containing 5 mmol/L 3-MA, and medium containing 10 *μ*mol/L AA with 5 mmol/L 3-MA, respectively. (b) HKC cells were transiently transfected with Beclin 1 siRNA or control siRNA. Afterwards, the experiments were divided into the following 4 groups: HKC cells in medium; HKC cells in medium containing 10 *μ*mol/L AA; HKC cells transfected with control siRNA in medium containing 10 *μ*mol/L AA; HKC cells transfected with Beclin 1 siRNA in medium containing 10 *μ*mol/L AA. After 12 h (for mRNA detection) and 36 h (for protein measurement) incubation, the mRNA and protein expression of fibronectin were determined by real-time quantitative PCR and Western blot assay, respectively. The relative protein expression level is expressed as the ratio of target protein/*β*-actin. Experiments were repeated 5 times independently. Values are represented as mean ± SD (*n* = 5). ^*∗∗*^*P* < 0.01 versus control group. ^Δ^*P* < 0.05, ^ΔΔ^*P* < 0.01 versus AA group. ^#^*P* < 0.05 versus AA + control siRNA group; ^##^*P* < 0.01 versus AA + control siRNA group.

Myofibroblasts derived from renal tubular epithelial cells by EMT can secrete ECM including fibronectin, which is involved in the renal interstitial fibrosis. To investigate the effects of autophagy on the expression of ECM in AA-treated HKC cells, the inducers and inhibitors of autophagy were used for the experiments, respectively. Results showed that, compared with AA group, rapamycin and SF medium both significantly upregulated the mRNA and protein expression of fibronectin (*P* < 0.05 or *P* < 0.01) (Figure 7), while 3-MA and Beclin 1 gene knockdown both significantly downregulated the mRNA and protein expression of fibronectin (*P* < 0.05 or *P* < 0.01) (Figure 8). These results suggest that induction of autophagy can significantly increase fibronectin expression, while inhibition of autophagy can significantly decrease fibronectin expression in AA-treated HKC cells.

## 4. Discussion

AAN includes any form of toxic interstitial nephropathy that is caused either by the ingestion of plants containing AA as part of traditional phytotherapies (formerly known as “Chinese herbs nephropathy”) or by the environmental contaminants in food (Balkan endemic nephropathy) [2]. Main pathological character of CAAN is renal interstitial fibrosis. Myofibroblasts in renal interstitium play a vital role in the fibrotic course by synthesis and secretion of ECM [5]. In renal interstitial myofibroblast pool, myofibroblasts from tubular epithelial origin undergoing EMT contribute 5% to 36% [21–23], and renal tubular epithelial cells participate in renal interstitial fibrosis in CAAN mainly through EMT [5, 24].

It was reported autophagy plays a critical role in kidney maintenance, diseases, and aging [13, 15]. Ischemic, toxic, immunological, and oxidative insults can cause an induction of autophagy in renal epithelial cells, which is involved in various kidney diseases [15]. However, role of autophagy in the kidney diseases is complex, since both the up- and downregulation of autophagy have been shown to be protective against different kidney diseases [15]. Therefore, further research is required to determine the specific contribution of autophagy to individual renal disease.

Recent studies also showed that autophagy was able to occur under AA stimulation, but the action of AA-induced autophagy on the AAN is not entirely clear [19, 20]. Zeng et al. [19] found that autophagy occurred earlier than apoptosis in the AA-treated rat renal proximal tubular epithelial cells (NRK52E), and the AA-induced autophagy could extenuate apoptosis. While Yang et al. [20] found that both autophagy and apoptosis occurred in the AA-treated NRK52E cells, the AA-induced autophagy could promote apoptosis, which is just the opposite of the finding of Zeng et al. However, the effects of autophagy on EMT in AA-treated HKC cells have not been studied so far.

In this research, the temporal dynamic changes of AA-induced autophagy and EMT in HKC cells were studied. We found that autophagy occurred prior to EMT, and when autophagy gradually attenuated after reaching the peak at 12th hour, EMT still continued to increase. Furthermore, the effects of inducers and inhibitors of autophagy on EMT occurrence in AA-treated HKC cells were also studied. We found that the inducers of autophagy, rapamycin [25], and SF medium [26], both significantly enhanced EMT at the same time of promoting autophagy, while the inhibitor of autophagy, 3-MA [27], and Beclin 1 gene knockdown both significantly attenuated EMT at the same time of weakening autophagy. In addition, myofibroblasts derived from renal tubular epithelial cells by EMT can secrete ECM, so the effects of inducers and inhibitors of autophagy on fibronectin production in AA-treated HKC cells were also researched. Results showed that the increase or decrease of fibronectin was consistent with the enhancing or weakening of EMT. So, the above research results suggest that the AA-induced autophagy can promote EMT and ECM production in HKC cells.

It is also found by other studies that autophagy could promote epithelial-mesenchymal transition including EMT in non-AA-treated renal tubular epithelial cells [28, 29]. Pang et al. [28] reported that TGF-*β*1 induced both autophagy and epithelial-mesenchymal transition in mouse tubular epithelial cells (C1.1 cells). Furthermore, 3-MA and Beclin 1 gene knockdown both reduced TGF-*β*1-induced epithelial-mesenchymal transition; rapamycin increased TGF-*β*1-induced epithelial-mesenchymal transition; and serum rescue-inhibited autophagy reversed epithelial-mesenchymal transition. In addition, Moon et al. [29] reported that endoplasmic reticulum stress inducers, tunicamycin (TM) and thapsigargin (TG), induced autophagy as well as EMT in human proximal tubular epithelial cells (HK-2 cells). Autophagy inhibitors, 3-MA and bafilomycin suppressed the TM- or TG-induced EMT, and Beclin 1 gene knockdown blocked the TM- or TG-induced EMT. Our study results and the above results all suggest that autophagy can promote EMT occurrence in renal tubular cells.

However, there is a study in which findings are contrary to the results of above studies including our study. Xu et al. [30] reported that high glucose induced both autophagy and EMT in human proximal tubular cells (HK-2 cells). Nevertheless, autophagy inhibitors, 3-MA and chloroquine diphosphate, and autophagy-related protein 5 gene (*Atg *5) knockdown all exacerbated EMT, while rapamycin attenuated EMT. It is unclear why the result of Xu et al.'s study is contrary to the results of above studies including our study. However, it has been known that autophagy has dual roles which can exert both beneficial and aggravating effects on diseases [31]. So, it is possible that the autophagy occurred in different environments (such as different stimuli and growth conditions) may pay different, even opposite, roles in EMT occurrence.

The molecular mechanism by which autophagy affects EMT has not been fully elucidated. Pang et al. [28] consider that TGF-*β*1-induced autophagy links *β*-catenin and Smad signaling to promote EMT in C1.1 cells through a novel pY654-*β*-catenin/p-Smad2/ILK pathway. Moon et al. [29] think that endoplasmic reticulum stress induced EMT through autophagy via activation of c-Src kinase in HK-2 cells. However, Xu et al. [30] did not report the mechanism how autophagy antagonizes EMT in the high glucose-treated HK-2 cells. It is very important to study the molecular mechanism of autophagy acting on EMT, which may also be one of the factors causing autophagy to produce a different effect on EMT. One limitation of the present study is that such a molecular mechanism has not been researched, which will be performed in our next work.

## 5. Conclusion

In conclusion, our research results suggest AA can induce both autophagy and EMT, and AA-induced autophagy can promote EMT occurrence and fibronectin production in AA-treated HKC cells. Our next research is to explore the molecular mechanism by which autophagy promotes EMT in the HKC cells stimulated with AA.

## Figures and Tables

**Table 1:** Primer sequences for real-time quantitative RT-PCR analysis.

Target	Primer sequence (5′-3′)
*α*-SMA	
Forward	GGGACGACATGGAAAAGATCTG
Reverse	CAGGGTGGGATGCTCTTCAG
E-cadherin	
Forward	GCCCCGCCTTATGATTCTCTGC
Reverse	CCTCGCCGCCTCCGTACATGTC
Beclin 1	
Forward	GATGGTGTCTCTCGCAGATTC
Reverse	CTGTGCATTCCTCACAGAGTG
Fibronectin	
Forward	AAGACACCTTCGGGGGAAATA
Reverse	GCAGAAAGTGTAAAGCTATCTCCAT
*β*-Actin	
Forward	GGAGCAATGATCTTGATCTTC
Reverse	CCTTCCTGGGCATGGAGTCCTG

**Table 2:** Primary and secondary antibodies for Western blot assay.

Primary antibody	Cat number	Dilution	Secondary antibody
Rabbit anti-Beclin-1 mAb (Cell Signaling)	#3495	1 : 1000	Goat anti-rabbit IgG secondary antibody (LI-COR Biosciences)
Rabbit anti-LC3 mAb (Sigma-Aldrich)	L7543	1 : 1000	Ditto
Mouse anti-E-cadherin mAb (Cell Signaling)	#14472	1 : 1000	Goat anti-mouse IgG secondary antibody (LI-COR Biosciences)
Mouse anti-*α*-SMA mAb (Sigma-Aldrich)	A2547	1 : 1000	Ditto
Mouse anti-*β*-actin mAb (Sigma-Aldrich)	A1978	1 : 1000	Ditto
Mouse anti-fibronectin mAb (Santa Cruz)	sc-8422	1 : 1000	Ditto
